# Evaluating patient-reported outcome measures (PROMs) for future clinical trials in adult patients with optic neuritis

**DOI:** 10.1038/s41433-023-02478-z

**Published:** 2023-03-17

**Authors:** Jesse Panthagani, Charles O’Donovan, Olalekan Lee Aiyegbusi, Xiaoxuan Liu, Susan Bayliss, Melanie Calvert, Konrad Pesudovs, Alastair K. Denniston, David J. Moore, Tasanee Braithwaite

**Affiliations:** 1 University Hospitals Birmingham, Birmingham, UK; 2 School of Immunology and Microbiology, King’s College London, London, UK; 3 Centre for Patient Reported Outcomes Research (CPROR), Institute of Applied Health Research, Birmingham Health Partners for Regulatory Science and Innovation, NIHR Birmingham Biomedical Research Centre, NIHR Applied Research Collaboration West Midlands, and NIHR Birmingham-Oxford Blood and Transplant Research Unit (BTRU) in Precision Transplant and Cellular Therapeutics, University of Birmingham, Birmingham, B15 2TT UK; 4 Institute of Inflammation and Ageing, University of Birmingham, University Hospitals Birmingham, Health Data Research UK, London, UK; 5 Institute of Applied Health Research, University of Birmingham, Birmingham, UK; 6 Centre for Patient Reported Outcomes Research (CPROR), Institute of Applied Health Research, Birmingham Health Partners for Regulatory Science and Innovation, NIHR, Birmingham Biomedical Research Centre, NIHR Surgical Reconstruction and Microbiology Centre, NIHR Applied Research Collaboration West Midlands, and NIHR Birmingham-Oxford Blood and Transplant Research Unit (BTRU) in Precision Transplant and Cellular Therapeutics, University of Birmingham, Birmingham, B15 2TT UK; 7 University of New South Wales, New South Wales, Australia; 8 Institute of Inflammation and Ageing, and Centre for Patient Reported Outcomes Research (CPROR), Institute of Applied Health Research, Birmingham Health Partners for Regulatory Science and Innovation, NIHR Birmingham-Oxford Blood and Transplant Research Unit (BTRU) in Precision Transplant and Cellular Therapeutics, University of Birmingham, University Hospitals Birmingham, Health Data Research UK, London, UK; 9 School of Immunology and Microbiology, King’s College London, and The Medical Eye Unit, Guy’s and St Thomas’ Hospital NHS Foundation Trust, London, UK

**Keywords:** Optic nerve diseases, Eye manifestations, Education

## Abstract

**Objective:**

To search for and critically appraise the psychometric quality of patient-reported outcome measures (PROMs) developed or validated in optic neuritis, in order to support high-quality research and care.

**Methods:**

We systematically searched MEDLINE(Ovid), Embase(Ovid), PsycINFO(Ovid) and CINAHLPlus(EBSCO), and additional grey literature to November 2021, to identify PROM development or validation studies applicable to optic neuritis associated with any systemic or neurologic disease in adults. We included instruments developed using classic test theory or Rasch analysis approaches. We used established quality criteria to assess content development, validity, reliability, and responsiveness, grading multiple domains from A (high quality) to C (low quality).

**Results:**

From 3142 screened abstracts we identified five PROM instruments potentially applicable to optic neuritis: three differing versions of the National Eye Institute (NEI)-Visual Function Questionnaire (VFQ): the 51-item VFQ; the 25-item VFQ and a 10-item neuro-ophthalmology supplement; and the Impact of Visual Impairment Scale (IVIS), a constituent of the Multiple Sclerosis Quality of Life Inventory (MSQLI) handbook, derived from the Functional Assessment of Multiple Sclerosis (FAMS). Psychometric appraisal revealed the NEI-VFQ-51 and 10-item neuro module had some relevant content development but weak psychometric development, and the FAMS had stronger psychometric development using Rasch Analysis, but was only somewhat relevant to optic neuritis. We identified no content or psychometric development for IVIS.

**Conclusion:**

There is unmet need for a PROM with strong content and psychometric development applicable to optic neuritis for use in virtual care pathways and clinical trials to support drug marketing authorisation.

## Abstract (zh)

目的: 寻找并严格评估在视神经炎中开发或验证的患者报告结局测量 (PROMs) 的心理测量质量, 以支持高质量的研究和临床诊疗。

方法: 我们系统地检索了MEDLINE(Ovid)、Embase(Ovid)、PsycINFO(Ovid)和CINAHLPlus(EBSCO), 以及截至2021年11月的其他文献, 以确定适用于与任何系统性或神经性疾病相关的成人视神经炎的PROM开发或验证的研究。我们将经典测试理论或Rasch分析方法开发的工具用于研究。我们使用既定的质量标准来评估内容开发、有效性、可靠性和响应性, 将多个领域从A (高质量) 到C (低质量) 分级。

结果: 从3142份筛选的摘要中, 确定了五种可能适用于视神经炎的PROM工具: 国家眼科研究所 (NEI) 视觉功能问卷 (VFQ) 的三个不同版本: 51-item VFQ;25-item VFQ和10-item神经眼科补充问卷;以及视觉障碍影响量表 (IVIS), 这是多发性硬化症生活质量清单 (MSQLI) 手册的组成部分, 来源于多发性硬化症功能评估 (FAMS) 。心理测量评估显示, NEI-VFQ-51和10 item神经模块有些相关的内容, 但心理测量发展较慢, 而FAMS使用Rasch分析法有较快的心理测量开发, 但只与视神经炎有一定的关联。我们没有发现IVIS的内容或心理测量开发。

结论: 目前对适用于视神经炎且具有强大内容和心理测量开发的PROM的虚拟诊疗路径和临床试验尚不多, 不足以支持药物上市授权。

## Introduction

Finding more effective treatments for rare diseases and inflammatory conditions, including optic neuritis (ON), is a research priority highlighted by stakeholders internationally [1, 2]. Whilst in the UK, optic neuritis is most strongly and frequently associated with Multiple Sclerosis (MS), a similar number of patients develop optic neuritis in association with other infectious and immune-mediated inflammatory diseases (IMIDs) combined [3]. The acutely sight-threatening and potentially irreversible nature of untreated non-MS optic neuritis, makes vital the early differentiation from MS-optic neuritis, for consideration of high dose corticosteroids. A substantial proportion of patients then follow a relapsing course, including those with Neuromyelitis Optica Spectrum Disorder (NMOSD), Myelin Oligodendrocyte Glycoprotein antibody-associated disease and neurosarcoidosis. These patients often need chronic steroid-sparing systemic immunosuppressives to reduce the risk of flares and progressive disability [4]. Beyond the vision impacts and side effects of treatment, ON has important psychological and social impacts. The unpredictable nature of ‘attacks’ makes it difficult for patients to gain a sense of control over their illness [5, 6]. Both ON and MS-ON disproportionately affect young adults, limiting daily activities in their most socioeconomically productive years [7]. Since there is currently no cure for MS, or the majority of other rare diseases associated with ON, treatments are directed at symptom alleviation, or reduction of relapse frequency [8]. Furthermore, active optic neuritis, although visually limiting, may not be readily apparent to others, thereby contributing to the sense of isolation experienced by many people with ON and MS-ON [9].

Visual acuity remains the most established outcome parameter used by regulatory agencies, including the Food and Drug Administration (FDA) when considering therapeutic efficacy of diseases involving the visual pathway. However, this metric’s limitations are recognised. There has been growing focus on patient-centred definitions of efficacy which capture the extent of an individual’s lived experience of their condition [10]. Better integration of the patient voice is advancing research priority setting, outcomes design, and routine clinical practice in medicine, and in neurology and ophthalmology specifically [11–14]. Patient-reported outcome measures (PROMs) facilitate quantitative capture of the subjectively experienced impacts of disease and its treatment (Table 1 Glossary) [15]. Vision-related PROMs focus on the symptoms and impacts generic to many different eye diseases and conditions, whilst health-related PROMs focus on symptoms and impacts on a person’s health more generally, and some disease-specific PROMs have been developed. PROMs are particularly useful when interventions reveal otherwise similar efficacy using traditional outcome measures, or when an intervention provides only a small clinical improvement, yet patients experience other benefits or harms [16]. To be useful, for drug marketing authorization [13, 17], or for integration in remote care pathways emerging from the COVID-19 pandemic, PROMs need to be targeted to the constructs of interest, possess sound psychometric performance properties (e.g. as assessed using Rasch Analysis or item response theory (IRT) models), and be valid, reliable, responsive and acceptable to users [17, 18]. Well-designed PROMs yield a precise, interval-scaled measure for each quality of life domain, which is amenable to quantitative statistical analysis, and thus of tremendous value to a variety of stakeholders within and beyond the clinical trial space, including patients and clinicians [19].

**Table 1:** Glossary of key terms.

Concept, process or tool	Definition
Patient reported outcome measure (PROM)	PROMs are sets of questions or ‘items’ which form an ‘instrument’ used to quantify the subjective impacts of disease or its treatment. They can be broadly split into generic, or disease-specific measures. Generic measures usefully support comparison of the health status of different disease groups, whilst disease-specific PROMs, including instruments focused on signs or symptoms, offer more sensitive measurement of change in health status for that disease.
Quality of life (QoL)	Health-related QoL is a multidimensional construct, including all domains in which a patient can be affected by a disease or its treatments. These typically include symptoms, daily activities, mental, social, emotional, convenience and economic impacts.
Classic test theory	Classic test theory (CTT) is a quantitative approach to test the reliability and validity of a scale. It considers the relationship between the expected score (or ‘true’ score) and observed score on any given measurement. The true score is one assumed to be that which would be obtained if there were no errors in measurement. It assumes that random errors (i.e. the difference between a true score and a set of observed scores on the same individual) are normally distributed (without measuring/testing this) and summary item responses are coded so that higher responses reflect more of the concept.
Rasch model	The Rasch Model measures latent traits (like difficulty with daily vision-related tasks) and provides an internally valid measure by allowing non-linear raw data to be converted to a linear scale, which then can be evaluated through the use of parametric statistical tests. It assumes that the probability of a given person/item interaction is governed by the difficulty of the item and the ability of the person, that are determined by the item locations on the presumed latent variable along with the rating scale structure.
Principal Component Analysis (PCA)	Principal Component Analysis (PCA) is a dimension-reducing tool that replaces the variables in a data set by a smaller number of derived variables.

The landmark Optic Neuritis Treatment trial, 30 years ago, explored a specific subgroup of ON patients aged 18 to 45 years with acute unilateral ON, with no known systemic disease (besides MS) [20]. This evidence base is predominantly applicable to MS-ON, and not to non-MS ON, which is responsible for over half of all incident ON in the UK [3]. Furthermore, a new corticosteroid treatment trial for optic neuritis has been proposed, addressing multiple limitations of the earlier trial [20, 21]. These include aspects of trial design, exploring the role of hyperacute steroid treatment, and use of more robust outcome measures aligned with contemporary clinical practice [21]. There is explicit need for a PROM able to capture treatment benefits and side effects across multiple quality of life domains [21]. This could shed important new insights for patient management in ON through the disease course. This systematic review aimed to identify and psychometrically evaluate the quality of PROMs developed for, or validated in, adults with optic neuritis, to consider whether any existing instruments meet the needs of a new trial. This review is part of a wider project informing the development of robust PROMs and item banks for use in ophthalmology [22, 23].

## Methods

The methodology followed our published PROSPERO protocol (CRD42019151652) [24]. The systematic review is reported in line with PRISMA guidance [24–26].

### Searches

We searched the following electronic databases on 11 November 2019, and updated the searches to 5 November 2021: MEDLINE (Ovid), Embase (Ovid), PsycINFO (Ovid) and CINAHL Plus (EBSCO). The search strategy combined index and free text terms for optic neuritis (and also, separately, scleritis and uveitis), and terms relating to quality of life, health status indicators or patient-reported outcomes, with no restrictions on the language or year of publication (see Supplementary Panel 1). The MEDLINE search strategy was adapted for use on all databases. We screened references of included studies, to identify additional instruments. Where multiple studies referenced the same PROM, we searched citations to obtain the study reporting the original PROM’s development and any subsequent revisions and reports relating to instrument quality appraisal or validation. Two reviewers (TB and JP) also independently searched a database maintained by the United States National MS Society to identify potentially relevant PROMs for optic neuritis [27].

### Study selection

We included studies reporting PROM content identification, development, psychometric assessment, or validation to assess the impact of optic neuritis in adult patients. We included optic neuritis of any cause, at any time from first presentation, and did not limit our search to demyelinating optic neuritis. We included broad search terms for patient-reported outcomes and ‘quality of life’, considering ‘quality of life’ as an umbrella term including multiple domains (see Table 1) [28]. We sought studies using disease-relevant content development methods such as structured/semi-structured interviews, focus groups and/or literature reviews, but did not exclude validation studies with weaker content development (e.g. based on expert opinion). We excluded editorials, reviews, conference abstracts and studies reporting instruments developed solely for use in children. We excluded studies reporting PROM use without development or validation, but searched the references of such studies to ensure capture of the original instrument’s development.

### Main outcomes

For each included study, we extracted study characteristics (publication year, citation, country/region, sample size) and characteristics of patients on whom the instrument was developed/assessed/validated. This included disease type(s) and subtypes, age, sex, ethnicity, and, if reported, the proportion of patients on systemic antimicrobial or anti-inflammatory therapy. We extracted the name of the PROM, the QoL domains covered, the number of items in each domain, and any subtypes of optic neuritis covered by the PROM.

### Data extraction, synthesis and analysis

Search results were uploaded to Endnote 20 (Clarivate Analytics). All titles and abstracts were screened by two independent reviewers (CO/TB and TB/XL), to remove irrelevant articles. Full text articles were obtained for studies that potentially met eligibility criteria. Abstracts that did not provide the reviewers with sufficient information to make a decision were taken forward for full-text screening, to minimise the risk of missing a potentially relevant article. At any stage, if the reviewers were unable to reach consensus, an additional reviewer was consulted (KP). Two reviewers (TB and OLA/JP/CO) independently extracted data from studies meeting the inclusion criteria, using a standardised form. We attempted to contact investigators for clarification where we were unable to grade elements not reported.

### PROM quality assessment

Two reviewers (TB and OLA/CO), with adjudication by a third (KP), considered the overall extent to which the instrument’s items were relevant to optic neuritis, based on the patient samples used for item identification and development, and for instrument validation. We graded relevance as very relevant, somewhat relevant, or not very relevant.

We assessed the quality of each PROM using established quality criteria (see Supplementary Table 1 definitions), adapted from the US Food and Drug Administration framework and guidelines [29], and COSMIN Standards for the selection of health status Measurement Instruments [30], grading each of multiple domains from A (high quality) to C (low quality) [31]. The framework has been used previously to appraise the quality of PROMs in ophthalmology [17, 32], including retinal disease [31], cataract [33], refractive surgery [34], refractive error [35], amblyopia and strabismus [36], and keratoconus [37]. We reviewed instrument content development, and appraised item identification and item selection. For item identification we assigned a grade ‘A’ for, “comprehensive consultation with patients,” if a sufficient number (i.e. more than 30) of relevant patients were included to achieve content saturation [38]. For item selection, we assigned a grade ‘A’, based on the COSMIN guidelines, if the pilot instrument contained more than seven times the number of patients than items in the instrument (or in the case of multidimensional instrument, seven times the number of items in the largest domain representing a unidimensional construct); if the patient sample was fewer than five times the number of items we graded this domain ‘inadequate’ (grade ‘C’) [39].

For instruments developed using classic test theory-based psychometric approaches, we assessed acceptability, item targeting and internal consistency, but we highlighted as a limitation that more modern psychometric approaches had not been considered (highlighting Table 2 cells in dark red to emphasise ‘not done’) [40]. For instruments developed using the more rigorous Rasch Analysis approach, we assessed response categories, dimensionality, measurement precision, item fit statistics, differential item functioning and targeting [19].

**Table 2:** Characteristics of included studies.

First author, year	Instrument name(s)	Domains/scales of QoL	Items	Optic neuritis relevant items^a^, *n*	Country	Patients, *N*	Patient characteristic	Completion time
Mangione, 2001 [42]	NEI VFQ-25	9 subscales: General health (1 item), general vision (1 item), near vision (3 items), distance vision (3 items), ocular pain (2 items), colour vision (1 item), peripheral vision (1 item), driving (3 items); and VISION-SPECIFIC social functioning (2 items), mental health (4 items), dependency (3 items), and role limitations (2 items).	26	25	USA	Data from *n* = 262 pilot study and *n* = 597 field test who completed an earlier 51-item instrument, for validation	*N* = 262 of which none had optic neuritis or optic nerve involving eye disease. Mean age 61 years, 54% female, 81% White.	Aimed to be 5 minutes, but not reported
Raphael, 2006 [41]	10-item NEI VFQ-25 Neuro-Ophthalmic supplement VALIDATION STUDY	In addition to NEI VFQ-25, 3 extra domains, vision function (6 items), visual functioning in daily life (2 items) and lid/eye appearance (2)	26 + 10	25 + 8	USA	215	Included *n* = 47 MS patients with acute optic neuritis.	Not reported
Cella, 1996 [5]	Functional Assessment of Multiple Sclerosis (FAMS)	The final 59 items comprising the six subscales: mobility (7 items), symptoms (7 items), emotional well-being (7 items); general contentment (7 items); thinking/fatigue (9 items), family/social well-being (7 items); unscored (15 items)	59	2, pain and headache	USA, two sites	20 MS patients with varying symptoms for item generation; 433 MS patients for development validation (377 from mail survey and 69 from clinical visit)	MS patients (*n* = 433) Average age 44.9 years (22–86); 70% women; 73% married and 90% white; mean education of 14 years (7–29 years). One-third of the patients were on disability. Relapsing-remitting disease (*n* = 194) and those with progressive disease (*n* = 174)	Not reported
MSQLI, 1997 [44]	IVIS	5 items. Difficulty: reading personal notes; printed letters; reading dials; watching TV; identifying house numbers street signs.	5	5	Not reported	Not reported	Not reported	2–3 minutes
Cole, 2000 [43]	National Eye Institute Visual Function Questionnaire (NEI–VFQ) VALIDATION STUDY in ON	51 items comprising of 14 subscales: overall health, overall vision, difficulty with near vision, difficulty with distance vision, limitations in social functioning, role limitations due to vision, dependency due to vision, mental health symptoms due to vision, future expectations for vision, driving difficulties, limitations with peripheral and colour vision, and pain or discomfort in or around eyes.	51	51	California, USA	244 MS patients	All patients with acute unilateral optic neuritis and no indication of a causal systemic disease other than MS were enrolled. Average age 40 (+/− 7); 215 (79%) were female and 215 (88%) were white.	Not reported

^a^based on our clinical judgement of whether questions look relevant to optic neuritis.

In both study types, we assessed validity (concurrent, convergent, discriminant and known group validity), reliability (test-retest) and responsiveness (see Supplementary Table 1 for definitions). Where the patient sample used to validate the instrument was not independent from the sample used to develop it (across one or more published papers) we highlighted this as a limitation of the instrument.

## Results

The systematic search of bibliographic databases and cited references identified 3876 records, reducing to 3412 after removal of duplicates. We identified three studies reporting differing versions of a vision disorder specific instrument, the National Eye Institute Visual Function Questionnaire, containing 25 items (NEI-VFQ-25), 51 items (NEI-VFQ-51), and a 10-item add-on module, validated for neuro-ophthalmic conditions (including MS-associated optic neuritis) [41–43].

**Fig. 1:**
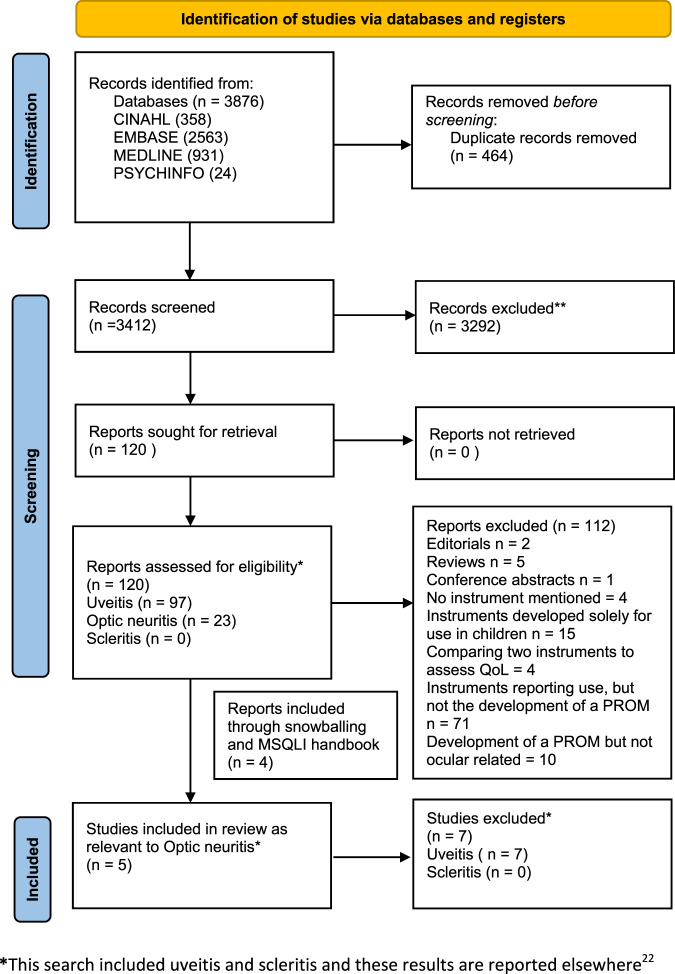
PRISMA flow diagram for systematic review.

Searching the National Multiple Sclerosis Society PROM database identified eight other instruments validated for use in MS, and two further included studies. We included one subscale from the Multiple Sclerosis Quality of Life Inventory, the Impact of Visual Impairment Scale (IVIS), which we understand was derived from the Functional Capacity Assessment, more commonly called the Functional Assessment of Multiple Sclerosis (FAMS) and referred to as FAMS from here on in [5, 44]. We excluded the remaining six instruments (see Supplementary Table 2 reporting MS PROMs) because of very limited coverage of items relevant to optic neuritis. The study selection process is presented in Fig. 1. Table 2 summarises the characteristics of the included studies. Table 3 summarises the findings comparing the psychometric quality appraisal of included studies against our predefined criteria (Supplementary Table 1). A justification of each grading assigned is available (Supplementary Table 2).

**Table 3:** Psychometric quality appraisal of included studies.

Instrument family	NEI-VFQ			IVIS	
First author, year	Raphael, 2006 [41]	Mangione 2001 [42]	Cole, 2000 [43]	MSQLI, 1997 [44]	Cella, 1996 [5]
PROM name	10-item Neuro-ophthalmology module (NOS-10)	NEI-VFQ-25 items	NEI-VF-51 items	IVIS	FAMS
Intended patient population instrument developed for	Neuro-ophthalmic	Vision impairment	Vision impairment	MS	MS
Item relevance to optic neuritis	Somewhat relevant	Not very relevant	Not very relevant	Not very relevant	Somewhat relevant
Independent development and validation samples?	Yes	No	Yes	Not reported	Yes
Item identification (for ON)	B	NR	NR	NR	NR^a^
Item selection	C	B	NR	NR	A^a^
Acceptability	NR	B	B	NR	A
Targeting	A	C	NR	NR	NR
Internal consistency	A	A	A	A	A
Response categories	NR	NR	NR	NR	A
Measurement precision	NR	NR	NR	NR	A
Dimensionality	NR	NR	NR	NR	A
Item fit	NR	NR	NR	NR	NR
DIF	NR	NR	NR	NR	B
Targeting	NR	NR	NR	NR	NR
Concurrent validity	A	A	C	NR	NR
Known group	A	A	A	NR	A^a^
Convergent	NR	NR	A	NR	A^a^
Discriminant	NR	NR	NR	NR	A^a^
Test-retest	NR	NR	NR	NR	A
Responsiveness	NR	NR	NR	NR	B

^a^note item identification, item selection, known group, development and validation was not specific to optic neuritis but to multiple sclerosis.

We excluded a study reporting preliminary development of a 46-item instrument in 15 patients with neuromyelitis optica (a cause of optic neuritis), as whilst a protocol for further instrument development was outlined, we could not find a manuscript reporting instrument completion, and did not hear from the authors following email enquiry [45, 46].

### National Eye Institute Visual Function Questionnaire (NEI VFQ-25)

The original NEI VFQ was developed between 1994 and 1998 for English-speaking adults aged ≥21 years with vision impairment from age-related macular degeneration, cataract, diabetic neuropathy, glaucoma or cytomegalovirus retinitis, following initial content development with multi-condition focus groups [47, 48]. A total of 262 patients were recruited from five academic centres, then a further 597 people were recruited in 1996 from multi-condition focus groups. The original 51-item instrument was developed from a 96-item pilot instrument, and took 15 minutes to administer. The shorter 25-item NEI VFQ-25 was developed in 2001 [42]. This included 11 vision-related subscales (general vision, near vision, distance vision, driving, peripheral vision, colour vision, ocular pain, vision-specific role difficulties, vision-specific dependency, vision-specific social functioning, and vision-specific mental health) and one general health item, with a few items per quality of life domain. Each subscale was scored by adding up ordinal values assigned to response categories (summary scoring) so that 0 represented the lowest and 100 the best possible score.

We graded the original NEI VFQ-25 development with ‘not done’ for item selection with respect to specific application to ON, though for its intended purpose as an eye disease-generic vision-specific tool it could be graded ‘A’. We scored NEI VFQ-25 ‘A’ for internal consistency based on classic test theory, but ‘B’ for acceptability and ‘C’ for targeting. However, with a single scale containing so many quality of life domains, with few items per domain, there is multidimensionality. This has been shown in Rasch analysis of NEI-VFQ data in other diseases [49]. Moreover, the purported 11 domains have been repeatedly shown to not be valid when tested using the Rasch model in other eye diseases. All four types of validity were assessed, but only concurrent and known group validity were graded ‘A’ in this tool’s capacity as an eye disease-generic vision-specific instrument, with convergent validity graded ‘B’ and discriminant validity graded ‘C’.

### NEI-VFQ 10-item Neuro-Ophthalmic Supplement (NOS-10)

Cleary et al. reported, “a questionnaire designed to assess the impact of an episode of optic neuritis on their quality of life,” six months after entry to the landmark 1991 Optic Neuritis Treatment Trial, which recruited patients with monocular acute ON [4]. The question set was completed by 87% (*n* = 382/438) patients [20]. We could not identify further detail in the literature on selection/development of question items. We reviewed the original 1991 ONTT case report form, which included questions on the earliest visual symptom, positive visual phenomena, presence, type and severity of pain in the affected eye, and a free text question on other ocular symptoms, but could not verify if these were the ‘questionnaire’ items [50]. Using the same questionnaire, Ma et al. later developed a 7-item MS-specific vision questionnaire (MSVQ) [20], for co-administration with the NEI VFQ-25 [51]. The instrument included six questions on vision (whether blurry, difficulty in bright sunlight, difficulty when eyes tired, two eyes see differently, trouble focusing on moving objects, binocular double vision), and one on vision-related functioning (difficulty using computer). No psychometric evaluation was used in the development of these two questionnaires, and it is possible the item content was selected by neurology or neuro-ophthalmology experts.

Raphael et al. subsequently reported validation of a 10-item Neuro-Ophthalmic supplement to the NEI-VFQ among 145 patients with MS including 47 patients who had a history of acute optic neuritis [41]. This supplement used the same content from the MSVQ (7-items) [20], along with three additional questions, selected from those items (including open questions and content from a questionnaire designed for patients following corneal surgery) [52], most frequently reported by a group of 80 MS patients to cause ‘slight difficulty’ (or worse) [41]. The three extra items included one additional question on vision-related functioning (difficulty parking car), and two on whether the eye/lid appearance was unusual, or ptosis was present, aiming to extend relevance of the instrument to patients affected by additional conditions such as myasthenia gravis.

Like the main NEI VFQ-25 instrument, in the validation study, items were presented using a categorical scale format, scored on a 0 to 100 scale. A composite score was calculated as the unweighted average of the 10 items. As there was no psychometric evaluation of items included in this instrument’s development, we graded this instrument ‘B’ for item identification and ‘C’ for item selection (no statistical justification provided), and considered it ‘somewhat relevant’ to optic neuritis. Content limitations aside, we graded ‘A’ for targeting, internal consistency, known group validity and concurrent validity. Other psychometric domains, including acceptability, responsiveness, repeatability and 2 other forms of validity were not reported.

### NEI-VFQ-51 validated in optic neuritis

Whilst the NEI-VFQ-51 was not developed for optic neuritis, Cole et al. reported validation of the original 51-item NEI–VFQ among 244 patients with acute unilateral MS-optic neuritis [43]. The questionnaire was administered as part of testing during an annual eye examination. The NEI-VFQ-51 included 14 subscales which were scored using a categorical scale, on a 0 to 100 scale (with 100 indicating highest function) [43]. The 14 subscales included overall health, overall vision, difficulty with near vision activities, difficulty with distance vision activities, limitations in social functioning due to vision, role limitations due to vision, dependency due to vision, mental health symptoms due to vision, future expectations for vision, driving difficulties, limitations with peripheral and colour vision, and pain or discomfort in or around eyes [43].

Item identification and selection were both graded as ‘not relevant’ as this study was seeking to validate a previously developed instrument. We graded acceptability as ‘B’ as the percentage of missing data in all subscales was below 40% (highest in difficulty with near vision activities at 10%). We graded internal consistency as ‘A’ as the average internal consistency over the 10 multi-item subscales (omitting the visual expectation subscale) was 0.86. We graded known group validity ‘A’, as there was a significant difference (*p* < 0.01) in NEI–VFQ Subscale Scores for distance activities, mental health, role difficulties, driving and peripheral vision in an independent subgroup. We graded construct validity ‘C’ as rank correlations between the NEI–VFQ subscales and the clinical vision tests ranged from small to modest. Other psychometric domains, including responsiveness, repeatability and discriminant validity were not reported.

### Impact of Visual Impairment Scale (IVIS)

The Impact of Visual Impairment Scale (IVIS) was reportedly derived from the Functional Assessment of Multiple Sclerosis (FAMS), developed by the Michigan Commission for the Blind. This instrument was only briefly outlined in the MSQLI handbook, without reference to a development or validation study. Therefore, we tried snowballing citations and searched PubMed between 1998 and 2006 for, ‘Impact of Visual Impairment Scale’ and ‘Functional Assessment’ and for all first authors who had published in MSQLI, but we were unable to identify further evidence of IVIS development or validation. We retained this instrument in our review but were unable to conduct a quality appraisal beyond the limited domains reported (without citation) in the MSQLI handbook.

The IVIS is a self-reported five-item instrument administered as a questionnaire or interview to provide an assessment of difficulties with simple visual tasks such as reading, watching television and recognising house numbers [44]. The MSQLI handbook reports the IVIS to have a reported Cronbach’s alpha of 0.86 (grade A), without detailing a study from which this derived [44]. In the original field testing of the MSQLI, the IVIS was reported to ‘significantly correlate’ with Visual item of the Kurtzke Functional Systems and with visual acuity (convergent and concurrent grade A, but we could not further verify this correlation) [44].

### Functional Assessment of Multiple Sclerosis (FAMS)

It was unclear where the five items in IVIS originated but we considered if possible they were informed by FAMS, as mentioned in the 1997 MSQLI handbook. We therefore reviewed the development and validation of FAMS published by Cella et al. [5]. The original 59-item FAMS instrument was developed from an 88-item pilot instrument. This included six subscales (mobility, symptoms, emotional wellbeing, general contentment, thinking/fatigue, and family/social well-being). Each subscale was scored on a five-category scale so that ‘0’ represented not affected and ‘4’ was very affected.

Initial content development for FAMS included a semi-structured interview with 20 MS patients and five MS specialists (yielding 135 new items), literature review and inclusion of 28 items from the Functional Assessment of Cancer Therapy, General version (FACT-G) and from the Fatigue Severity Scale developed by the Department of Neurology at the University of Chicago. Items were winnowed down to the 88-item pilot instrument. In the development and validation study, a total of 433 patients with MS were recruited from two hospitals in Chicago, USA. Of these 377 (74% completion rate) participated via postal survey, and the remaining 56 (81.2% completion rate) patients participated during a clinic visit, with the latter group completing additional validation tests (completing the Kurtzke Extended Disability Status Scale (EDSS) and the Scripps Neurological Rating Scale (NRS)), and test-retest reliability 3-7 days later [53, 54].

We graded the original FAMS ‘A’ for item identification and item selection in its intended purpose, as an MS-specific tool. We graded this tool as ‘somewhat relevant’ to optic neuritis, as although the domains addressed included mobility and emotional well-being which are important in ophthalmic quality of life, the study mentioned no information on the history or time course of optic neuritis in the 20 MS patients interviewed during item generation. Treatments and vision levels were also not reported. The content of FAMS may be driven by the quality of life impacts of MS, which are many and varied, only a subset of which are likely relevant to ON.

Instrument development was fairly strong, using responses from 377 MS postal survey patients and principal component analysis (PCA), to identify 63 items in five distinct ‘factors’ or subscales with identifiable conceptual meaning (accounting for 47.7% of the total variance) [55]. Prior to Rasch analysis, one of the factors (emotional wellbeing) was divided into two subscales for conceptual and practical reasons. Rasch analysis using BIGSTEPS further refined and developed the instrument into 44 items in six unidimensional sub-scales. The full data and outputs from the Rasch analysis were not reported in the manuscript, limiting complete quality appraisal. An additional 15 unscored items were also retained in the final 59-item instrument, ‘based on their potential clinical and empirical value’, which was a quality limitation.

Both CTT and limited Rasch metrics were reported for FAMS. We scored FAMS ‘A’ for internal consistency, with Cronbach’s alpha coefficients reported as universally high (range 0.82 to 0.961), and ‘A’ for measurement precision, response categories and dimensionality. We graded test-retest repeatability ‘A’, as the reliability coefficients ranged from 0.85 to 0.91. Independent validation data was available for 56 patients in whom the instrument was not developed, and was of excellent quality with many different instruments included to explore three aspects of validity. Whilst the investigators reported that concurrent validity was assessed, we did not find a clinical measure (defined in the quality appraisal criteria used in our review) against which the instrument was assessed and so graded this ‘not reported’. We were unable to assess external generalisability as the patients affected by MS-optic neuritis differ from optic neuritis generally.

## Discussion

To our knowledge, this is the first systematic review to appraise the psychometric quality of PROMs developed for and/or validated in optic neuritis. The review highlights a relative paucity, especially of tools developed or validated for application in non-MS ON. Psychometric appraisal revealed the 10-item neuro module to supplement the NEI-VFQ-25 had some relevant content development, some validation, but slightly limited psychometric development by contemporary standards. The FAMS had stronger psychometric development and stronger validation and reliability assessment, but content development may have been only somewhat relevant to MS-ON. In addition, this study did not report subgroup analysis exploring whether clinical differences between patients with and without optic neuritis (e.g. visual acuity, contrast sensitivity, visual field loss and/or scotomas) influenced responses (i.e. differential item functioning). We identified no published content or psychometric development or validation for IVIS.

There is need for a robust PROM applicable to both MS-optic neuritis and non-MS optic neuritis and their treatments, to inform future care, and to support virtual patient monitoring and new trials [12]. Our quality appraisal highlighted multiple weaknesses. The primary limitation of most available PROMs for ON (NEI-VFQ and IVIS) is that they were developed prior to the now widespread use of psychometric development approaches based on Item Response Theory. Petrillo and colleagues have outlined multiple issues with using classic test theory for psychometric evaluation [56]. Specifically, analysis is not based on interval-level measurement but on counts (summary scores of items), findings are dependent on the scale and sample, missing data cannot be handled easily, and the standard error of measurement around individual patient scores are assumed to have a constant value. Contemporary psychometric tools, such as the Rasch model, permit more robust examination of validity and interpretability. For example, multiple studies have psychometrically evaluated the NEI-VFQ-25 in patients with different ocular conditions and the general population, and have identified major shortcomings with respect to reliability, validity and dimensional structure [42–46]. Exploring data from 2487 patients with retinal disease, Petrillo et al. reported that the NEI-VFQ-25 contained disordered response thresholds (15/25 items) and mis-fitting items (8/25 items) [47, 57]. The psychometric performance has been similarly critiqued in low vision and cataract populations, with studies identifying only two unidimensional scales individually fitting the Rasch model [44, 45]. A Rasch re-engineered NEI-VFQ with two domains and fewer items has been developed [45, 47], but has not been validated in ON. The NEI-VFQ-25 remains widely used as a secondary outcome measure in ophthalmic clinical trials, and in ON trials specifically, including the 10- and 15-year follow-up studies of the 1991 Optic Neuritis Treatment Trial (*n* = 319) [58], and the more recent RENEW trial of Opicinumab (*n* = 82) [59, 60].

The FDA have noted the lack of validated PROMs in ophthalmology, and indicated that PROMs developed and validated using Rasch Analysis approaches would be required for the high-stakes situation of a pharmaceutical labelling claim [61]. The PROMs identified in our review were also developed and validated many years before the widespread application of COSMIN guidelines and Rasch Analysis-based quality appraisal tools. For example, only one included study reported on differential item functioning (DIF) [5]. Even with stronger psychometric analysis approaches able to explore DIF than were available decades ago, there is still unmet need for detailed and transparent reporting on item measures in relation to the specific sample of persons chosen to participate in a given study, as the different effects of different types of visual impairments (near and distance visual acuity, colour, contrast sensitivity and field of vision), or comorbidities, on item responses may violate the Rasch requirement of homogeneity of variance in measurement uncertainty. Clear reporting on missing data is also needed to permit appraisal of any potential risk of bias resulting from model artefact. For example, under the assumptions of Classic Test Theory or Lickert scoring, missing data in the raw scores (for example where the participant selects “not applicable” or “don’t do for reasons other than my vision”) cause distortion in the summary variable. Similarly, missing data may lead to disordered response thresholds, generated as an artefact of the model employed (eg the partial credit model or Andrich rating scale model).

A further general theme emerging from this review was very limited content development for ON. We could not identify how many patients with optic neuritis were consulted in the development and selection of the items which went on to be included in the PROMs. Typically, the COSMIN guidelines suggest that in order to develop a structurally valid PROM, at least seven times the number of relevant patients as the number of unidimensional items being assessed for inclusion are needed to develop ‘very good’ content; whereas if the patient sample is fewer than five times the number of items in the instrument, this is ‘inadequate’ [39]. To aim for a disease-specific PROM for every medical condition would be both unachievable and undesirable. Whilst there is likely to be very major overlap in the vision-related impacts of different eye diseases, if using the NEI-VFQ-25 in a clinical stakes trial, it may be useful to first validate the assumption that content from the original patients, who had one of just six eye diseases (age-related macular degeneration, cataract, diabetic neuropathy, glaucoma or cytomegalovirus retinitis) yields necessary and sufficient vision-related quality of life insights for the eye disease under investigation. PROMs developed without comprehensive content identification (saturation) are unlikely to have adequate external generalisability to other settings (different countries, demographics, disease subtypes and treatments), limiting translation into clinical practice. Of note, the quality appraisal criteria (Supplementary Table 1) themselves do not account for whether the patients included in content development were relevant to the outcome of interest for which their quality is being appraised. We therefore added an additional item pertaining to ‘relevance to ON’ in our appraisal process.

Also evident was a historic desire for short instruments with completion times around five minutes to minimise participant burden, in the context of clinical trial examination protocols. Quality appraisal indicates that this focus on speed may have come at the cost of psychometric instrument performance. Evidence suggests there are at least 10 domains of quality of life relevant to people with ophthalmic disease, extending beyond, but including symptoms of disease (see Table 1) [23]. Each domain of interest needs to be measured with a sufficient number of items, spread out on an interval scale, to yield a precise measure for that domain. This is impossible when only one item is included per scale, and the measure is likely to have low precision and reliability when only a few items are included per domain. Fortunately, the advent of computer adaptive testing offers a solution to the ‘time burden’ problem [62].

These multiple limitations may explain the historically poor uptake of PROMs in MS and ON clinical trials, in spite of the FDA encouraging incorporation of PROMs into clinical trials for over a decade [29]. For example, FAMS (developed 1996) was not included as a primary or secondary outcome measure in key phase III clinical trials for drugs which gained subsequent FDA approval for MS, including the AFFIRM trial of natalizumab, CARE-MS trial of alemtuzumab, INFORMS trial of fingolimod or DEFINE trial of dimethyl fumarate [63–66]. There have been some exceptions. However, where older non disease specific PROMs, developed using CTT, have been included in trials they have methodological limitations and have failed to find any significant differences. For example, Jacobs et al. included the Sickness Impact Profile (SIP) as a secondary outcome measure in the phase 3 study of recombinant interferon beta-la as treatment for relapsing-remitting MS [67]. Whilst the physical component summary score of the Medical Outcomes Study 36-Item Short-Form Health Survey (SF-36) was included as a secondary outcome in the ORATARIO phase 3 trial assessing the impact of intravenous ocrelizumab in primary progressive MS [68]. We hypothesise that the lack of differences detected at the person level may be due to the lack of disease-specific content, or the lack of precision of the summary scoring approach or both.

There has been variable uptake of PROMs into clinical practice, despite enthusiastic support from stakeholders and patient advocacy organizations [69]. A common theme has been historic emphasis on ‘hard’ outcomes, such as relapse rates or radiological features as surrogate markers of disease progression, particularly in MS trials. Reliance on such objective outcomes has been understandable but they may miss important aspects of morbidity [70]. In addition, clinical trial results reporting often does not provide PROM interpretation guidelines, which may exacerbate a sense of mystery around PROMs that does not exist for other outcomes [70].

### Strengths and limitations

We adhered to sound systematic review methodology including a comprehensive search for published PROMs and robust quality appraisal of identified instruments. We did not extensively search the grey literature or conference abstracts and may have overlooked reports of unpublished PROMs. We consider it unlikely that this would have resulted in the identification and inclusion of any high-quality PROMs not identified through the main search. Our search strategy included optic neuritis of any cause, but we did not conduct a separate search for all immune-mediated inflammatory diseases with which ON may be associated, and may therefore have overlooked some sets of relevant questions.

We felt the quality criteria we used (Supplementary Table 1), were limited in not holding studies utilising older and more simple classic test theory approaches to the same level of account in the grading scheme as studies developed using more modern Rasch Analysis approaches with principal components analysis [29–31, 71]. It is worth noting that not all the quality assessment criteria in Supplementary Table 1 are of equal value and importance. The possession of interval scaling and Rasch validity (especially precision and uni-dimensionality) is more important than assessments of validity, reliability, or acceptability. The criteria also did not require assessment of whether or not the patient samples used to develop and to validate a PROM were independent, which is important. We added these and recommend them as a modification to the grading criteria.

### Implications

The lack of methodologically robust PROMs in optic neuritis is a significant problem for multiple reasons. The recent coronavirus global pandemic has ushered in a period of accelerated service transformation in health systems internationally. This is driving major shifts towards virtual review and remote monitoring and in this context, PROMs could have an important role to play. PROMs improve patient satisfaction with care, symptom management, quality of life and survival rates [72]. The integration of PROM data through technological infrastructure has progressed rapidly leading to the incorporation of internet-based applications, touchscreen tablets and electronic health records [73]. For clinicians, PROM collection has been shown to enhance shared decision making by allowing the clinicians to better understand the patient’s symptoms and the impact on their quality of life. Furthermore, it can enhance workflow efficiency and save time when used regularly, e.g. by using the limited clinic time to explore a particular symptom burden highlighted from the instrument [74].

The potential value of using a PROM with strong psychometric performance as a trial endpoint cannot be understated. Not only do these permit alignment with the outcomes that most matter to patients, but there are major resource implications. Narrow standard errors around an outcome measure permit recruitment of smaller samples, with major cost saving for trial funders. Based on our quality appraisal, we are not able to recommend any of the currently available PROMs for therapeutic trials in optic neuritis.

### Future research

Further research to develop robust PROMs for optic neuritis is needed. Adherence to best practice in PROMs development (as described in guidance from the FDA) will support development of more robust, sensitive PROMs [75]. Larger samples of patients are generally needed for content identification and instrument development than have been used in the PROMs reported here. Future studies could aim for independence of development and validation samples, and recruit a sufficient sample size (>7x patients than number of items in largest unidimensional scale) for robust psychometric development using the Rasch Analysis approach. Transparent reporting on any differential item functioning by potentially relevant clinical characteristics or condition or disease co-morbidities is also needed. Investigators may find the PROTEUS, SPIRIT-PRO and CONSORT-PRO guidelines on the selection and reporting of PROMs for clinical trials helpful [75–78].

## Conclusion

This systematic review highlights an important, unmet need for the development and validation of PROMs that are able to measure the impact of optic neuritis, and its treatment, on multiple domains of quality of life. Demand for robust PROMs is anticipated to rise as not only patients and clinicians [74], but regulators, payers, accreditors, and professional organisations recognise their potential value [73]. Given the time and cost taken to develop a new PROM, and the increasingly important role for PROMs both in clinical trials and the modern health service, further research is needed to identify novel ways to reduce the multiple barriers to their development and wider generalisability. This will be essential to capture the quality of life outcomes that really matter to people.

## Supplementary information

Supplemental Information
