# Supplementary material for: Evaluating patient-reported outcome measures (PROMs) for future clinical trials in adult patients with optic neuritis
Source: Eye (Lond). 2023 Mar 17;37(15):3097–107. doi: 10.1038/s41433-023-02478-z (PMC10022552; doi:10.1038/s41433-023-02478-z)
Supplement: Supplementary file 1 — Supplemental Information [file 41433_2023_2478_MOESM1_ESM.docx]

**Online** **supplementary files**

**Supplemental Panel 1: Search strategy for MEDLINE**

Database: Ovid MEDLINE(R) <1946 to 5 November 2021>

1. quality of life/

2. health status/

3. health status indicators/

4. quality of life.tw.

5. health status.tw.

6. (hql or hqol or hrql or hrqol or qol).tw.

7. (symptom$ adj4 score$).tw.

8. (pros or pro or proms or patient-reported or health related or patient engage$ or patient satisfaction or PROM or patient reported).ti,ab.

9. or/1-8

10. exp Uveitis/

11. uveitis.ti,ab.

12. (inflam* adj3 sclera).ti,ab.

13. exp Scleritis/

14. scleritis.ti,ab.

15. exp Optic Neuritis/

16. optic neuritis.ti,ab.

17. or/10-16

18. 9 and 17

*****This search included uveitis and scleritis and these results are reported elsewhere^22^

**Supplemental Table 1: Quality appraisal framework for included studies**

| Property | Definition | Grade | Quality criteria |
| --- | --- | --- | --- |
| Content development | | | |
| Item identification | Identification of the initial item content | A | Comprehensive consultation with patients and literature review |
|  |  | B | Minimal consultation with appropriate patients, expert opinion and literature review |
|  |  | C | No consultation with patients |
| Item selection *on a sufficient sample* | Selection of items in the final instrument | A | A pilot instrument developed and tested with Rasch or factor analysis, statistical justification provided for removing items, removal of items with floor and ceiling effects, and consideration of the amount of missing data to obtain final item set. |
|  |  | B | Only some of the above techniques used |
|  |  | C | No pilot instrument, or statistical justification of items included in final instrument |
| Classic Test Theory (CTT)-based psychometric properties | | | |
| Acceptability | The percentage of missing data for each item and percentage of people for whom a PROM score can be computed | A | < 5 % |
|  |  | B | 5 % to < 40 % |
|  |  | C | > 40 % |
| Item targeting | PROM instrument scores should span the entire range; floor (proportion of sample at maximum score) and ceiling (proportion of sample at minimum score) effects should be low. | A | Either floor or ceiling effect < 5 % |
|  |  | B | Either floor or ceiling effect 5 % to < 40% |
|  |  | C | Either floor or ceiling effect < 40% |
| Internal consistency | The extent to which all the items of a test measure the same latent variable. Internal consistency is usually measured with Cronbach’s alpha, a statistic calculated from the pairwise correlations between items. Internal consistency ranges between negative infinity and one. | A | > 0.7 to < 0.95 |
|  |  | B | > 0.6 to < 0.7 |
|  |  | C | < 0.6 |
| Rasch-based psychometric properties | | | |
| Response categories | The extent to which the categories used to rate the items are chosen in a logical order (ordered categories). Evenly spaced categories (distance between category thresholds are expected to be between >1.40 and <5.00 logits). | A | All the categories were ordered, or ordering was obtained after repairing disordered categories, and evenly spaced categories |
|  |  | B | All the categories were ordered, or ordering was obtained after repairing disordered categories, but categories were not evenly spaced |
|  |  | C | Unrepairable, disordered categories |
| Dimensionality | The extent to which the instrument measures a single underlying construct. Principal components analysis (PCA) of the residuals based on 2 parameters: the amount of raw variance explained by the measure and eigenvalue of the unexplained variance in the first contrast or  PCA/t test protocol: % of t-tests of person measures obtained from the two item sets grouped based on PCA guided residual loading (first set => +0.3 and second set = < -0.3) significantly different/% of t-tests falling outside the range +/- 1.96 (95% CI). | A | Variance explained by the measure > 60% and eigenvalue of the first contrast < 2.0, or < 5% of the person estimates are significantly different, or % t tests falling outside 95% CI is < 5 % |
|  |  | B | Variance explained by the measure > 50% to < 60% and eigenvalue of the first contrast < 2.0, or > 5% to < 10% of the person estimates are significantly different, or % t tests falling outside 95% CI is > 5 % and < 10 % |
|  |  | C | Variance explained by the measure < 50%, eigenvalue of the first contrast > 2.0, indication of subsets of items (indicating multidimensionality) or > 10% of the person estimates are significantly different, or the lower bound of a Binomial 95% CI of the observed proportion overlaps >10 % |
| Measurement Precision | The extent to which an instrument distinguishes between different levels of participants’ abilities. Represented by person separation index or reliability coefficient (minimum acceptable value, separation = 2.0, or reliability α= 0.80) | A | > 2.50, α > 0.85 |
|  |  | B | 2.0 to 2.49, α > 0.80 to < 0.85 |
|  |  | C | < 2.0, α < 0.80 |
| Item fit statistics | The extent to which the items in the instrument fit with the Rasch model expectation. Two fit statistics, infit and outfit mean square, should have a value of 1 (acceptable range 0.50-1.50). | A | All items with infit and outfit mean square between 0.7 and 1.3 (or) infit and outfit standardized residuals < 2 |
|  |  | B | One or 2 items within the 0.5 to 1.5 range, or infit and outfit standardized residuals > 2.5 |
|  |  | C | More than 2 items outside the 0.5 and 1.5 limit or infit and outfit standardized residuals > 2.5 |
| Differential item functioning | The extent to which the levels of response ability of different subgroups of the same study population differ to an item (magnitude < 0.50 logits: insignificant, 0.50-1.0 logits: mild, > 1.0 logit: notable) | A | All items with DIF < 0.50 logits |
|  |  | B | Some items 0.50 to 1.0 logits and one at the most > 1.0 logits |
|  |  | C | More than one item > 1.0 logits DIF |
| Targeting | The extent to which item difficulty matches with the level of participants’ visual abilities. It is the difference between item and person means (difference of >1 logit indicates significant mistargeting). | A | < 1 logits |
|  |  | B | >1 to < 2 logits |
|  |  | C | > 2 logits |
| Validity | | | |
| Concurrent validity | The extent to which the instrument score correlates with the score of the clinical measure (e.g. visual acuity) | A | Tested against appropriate clinical measures and correlates 0.3 to 0.9 |
|  |  | B | Tested against debatable clinical measures and correlates 0.3 to 0.9 |
|  |  | C | Tested and correlates <0.3 or >0.9 |
| Convergent validity | The extent to which the instrument correlates with an existing instrument measuring a similar construct | A | Tested against appropriate instrument and correlates 0.3 to 0.9 |
|  |  | B | Tested against debatable instrument and correlates 0.3 to 0.9 |
|  |  | C | Tested and correlates <0.3 or >0.9 |
| Discriminant validity | The extent to which the instrument correlates with an existing instrument measuring a different construct | A | Tested against appropriate instrument and correlates <0.3 |
|  |  | B | Tested against debatable instrument and correlates <0.3 |
|  |  | C | Tested and correlates >0.3 |
| Known group validity | The extent to which the instrument can discriminate between clinically distinct groups | A | Tested in appropriate groups and significant difference between groups |
|  |  | B | Tested in debatable groups and significant difference between groups |
|  |  | C | Tested and nonsignificant difference between groups |
| Reliability | | | |
| Test-retest reliability | The extent to which the instrument demonstrated temporal stability when administered in 2 different periods. Intraclass correlation (ICC) > 0.8 is considered good | A | ICC > 0.8 |
|  |  | B | ICC 0.79 to < 0.60 |
|  |  | C | ICC < 0.60 |
| Responsiveness | | | |
| Responsiveness | The extent to which the instrument can detect clinically important changes over time (minimal important difference, which is the smallest difference in score which a patient perceives to be beneficial) | A | Change in score shown (increase or decrease) to have statistical significance |
|  |  | B | Instrument tested for responsiveness, but statistical significance not reported |
|  |  | C | No change in the PRO instrument score from baseline or statistically insignificant |
| KEY: A, high; B, medium; C, low; NR, not reported | | | |

**Supplemental Table 2: Table providing justification of the assigned quality appraisal gradings**

| **Instrument and first author** | | |
| --- | --- | --- |
| **NEI VFQ-25, Raphael et al 2006** | | |
| **Property** | **Grade** | **Explanation for grade** |
| Item identification | B | "The 10-Item Neuro-Ophthalmic Supplement was designed previously by our research group by survey and focus-group methods in patients with MS, ocular myasthenia gravis, and other conditions that cause diplopia (Tamhankar MA, abstract, presented at the North American Neuro-Ophthalmology Society Annual Meeting, March 2003).” “However, patients with MS and other neuro-ophthalmologic disorders, particularly those disorders that cause double vision and eye movement abnormalities, were not included systematically in the focus groups or study cohorts that were used to derive content for the NEI-VFQ-25." |
| Item selection | C | 10-item preliminary instrument administered to 215 patients (including 47 MS patients with a history of acute optic neuritis). 47 patients/10 items = 4.7 |
| Acceptability | NR |  |
| Item targeting | A | "For composite scores, percentages at the ceiling were 2% for the NEI-VFQ-25& supplement for patients. No patients had scores of 0 (floor) for either composite." |
| Internal consistency | A | "Cronbach score for the 10-Item Neuro-Ophthalmic Supplement was 0.85" "The overall Cronbach statistic for the NEI-VFQ-25 was 0.96; adding the 10-Item Neuro-Ophthalmic Supplement to the NEI-VFQ-25 resulted in persistently high levels of internal consistency reliability (Cronbach score, 0.96) in this cohort of patients with neuro-ophthalmologic disorders." |
| Response Categories | NR | Instrument not developed using Rasch Analysis |
| Dimensionality | NR | Instrument not developed using Rasch Analysis |
| Measurement precision | NR | Instrument not developed using Rasch Analysis |
| Itemfit statistics | NR | Instrument not developed using Rasch Analysis |
| Differential item functioning | NR | Instrument not developed using Rasch Analysis |
| Targeting | NR | Instrument not developed using Rasch Analysis |
| Concurrent validity | A | "Rank-correlations of monocular visual acuities with questionnaire scores revealed slightly higher correlations with worse eyes (rs = 0.35 for 10-Item Supplement; rs = 0.40 for NEI-VFQ-25 +Supplement) compared with better eyes (rs = 0.27 for 10-Item +Supplement; rs = 0.31 for NEI-VFQ-25 +Supplement)." |
| Known group validity | A | " Compared with the composite score for the NEIVFQ-25 alone, the combined score for the NEI-VFQ-25 + Supplement demonstrated a greater capacity to distinguish patients with neuro-ophthalmologic disorders from disease-free control subjects." "Accounting for age and gender, odds ratios in favour of participants with worse questionnaire scores being patients (vs disease-free control subjects) were greater for the NEI-VFQ-25 Supplement (odds ratios in favour of participant being a patient if questionnaire score was worse by 10 points: 4.4; 95% confidence interval (CI), 2.5, 7.9) compared with the NEI-VFQ-25 alone (odds ratio, 3.9; 95% CI, 2.2, 7.2; P.001 for all regression models)." |
| Convergent validity | NR |  |
| Discriminant validity | NR |  |
| Test-retest repeatability | NR |  |
| Responsiveness | NR |  |
| **NEI VFQ-25, Mangione et al 2001** | | |
| **Property** | **Grade** | **Explanation for grade** |
| Item identification | NA | Item content was obtained from patients with cytomegalovirus retinitis, age-related macular degeneration, cataract, diabetic retinopathy and glaucoma only, with no optic neuritis patients included |
| Item selection | B | "51-item NEI VFQ completed by two separate samples (1st consisting of 262 persons from 5 academic centres who participated in the 1994 pilot test of the NEIVFQ (pilot study), and the second sample consisted of 597 persons from the 1996 NEI VFQ Psychometric Field Test". No patients had optic neuritis. |
| Acceptability | B | Missing data for each item ranged from 0 to 37% |
| Item targeting | C | Ceiling effect was >40% in several scores |
| Internal consistency | A | "Internal consistency estimates for the NEI VFQ-25 subscales ranged from 0.71 to 0.85" |
| Response Categories | NR | Instrument not developed using Rasch Analysis |
| Dimensionality | NR | Instrument not developed using Rasch Analysis |
| Measurement precision | NR | Instrument not developed using Rasch Analysis |
| Item fit statistics | NR | Instrument not developed using Rasch Analysis |
| Differential item functioning | NR | Instrument not developed using Rasch Analysis |
| Targeting | NR | Instrument not developed using Rasch Analysis |
| Concurrent validity | A | "Correlations between responses on the NEI VFQ-25 and ETDRS visual acuity were in the range of 0.65 to 0.70 for subscales that reflected degree of difficulty with visual activities related to general vision, near vision, and distance vision'" "Visual field loss scores had moderate statistically significant correlations with the NEI VFQ-25 composite score, general vision, distance vision, near vision, peripheral vision, social functioning, dependency, and mental health subscales." |
| Known group validity | A | "Adjusted mean (SEM) scores for the NEI VFQ-25 near and distance vision scores for participants in the reference group vs those with age-related macular degeneration were statistically significant. Finally, adjusted mean (SEM) scores for the NEI VFQ-25 peripheral vision question for reference group members vs those with glaucoma were 97 and 76. All of these differences in mean scores were statistically significant. These selected comparisons provide evidence of between-group validity for the NEI VFQ-25". No groups included optic neuritis. |
| Convergent validity | NR |  |
| Discriminant validity | NR |  |
| Test-retest repeatability | NR |  |
| Responsiveness | NR |  |
| **NEI-VFQ, Cole 2000** | | |
| **Property** | **Grade** | **Explanation of grade** |
| Item identification | **NR** | **This was a validation study, not a development study** |
| Item selection | NR | **This was a validation study, not a development study** |
| Acceptability | B | There was missing data in all 13 subscales, this was <10% and was highest in near activities (25/244 or 10.2%) and lowest in general health (1/244 or 0.004%). |
| Item targeting | NR |  |
| Internal consistency | A | “The average internal consistency reliability over the 10 multi-item subscales (omitting the visual expectation subscale) was 0.86. The vision-specific expectation subscale was 0.46.” |
| Response Categories | NR | Instrument not validated using Rasch Analysis |
| Dimensionality | NR | Instrument not validated using Rasch Analysis |
| Measurement precision | NR | Instrument not validated using Rasch Analysis |
| Item fit statistics | NR | Instrument not validated using Rasch Analysis |
| Differential item functioning | NR | Instrument not validated using Rasch Analysis |
| Targeting | NR | Instrument not validated using Rasch Analysis |
| Concurrent validity | C | ‘Rank correlations between the NEI–VFQ subscales and the clinical vision tests ranged from small to modest. All NEI-VFQ subscales measure <0 with visual acuity with the exception of ocular pain which measures 0.04 with visual acuity.’ |
| Known group validity | A | “ NEI–VFQ Subscale Scores when compared with an Independent Reference Group (who were disease free) showed p<0.01 in distance activities, mental health, role difficulties, driving and peripheral vision. And p <0.05 in near activities, social functioning colour vison ” |
| Convergent validity | A | “The 25-item abbreviated version of the NEI–VFQ correlated well with the full 51-item field test version. Spearman rank correlations between the two versions were as follows: general health = 0.94; general vision = 0.84; near activities = 0.96; distance activities = 0.95; social functioning = 0.96; mental health = 0.92; role difficulties = 0.96; dependency = 0.88; driving = 0.90. The ocular pain, colour, and peripheral vision subscales are equivalent on the two versions.” |
| Discriminant validity | NR |  |
| Test-retest repeatability | NR |  |
| Responsiveness | NR |  |
| Independent development and validation samples | NR |  |
| **IVIS, MSQLI Handbook, 1997** | | |
| **Property** | **Grade** | **Explanation for grade** |
| Item identification | NR |  |
| Item selection | NR |  |
| Acceptability | NR |  |
| Item targeting | NR |  |
| Internal consistency | A | “The IVIS has a Cronbach's alpha of 0.86.” We were not able to identify the development study to confirm this. |
| Response Categories | NR |  |
| Dimensionality | NR |  |
| Measurement precision | NR |  |
| Item fit statistics | NR |  |
| Differential item functioning | NR |  |
| Targeting | NR |  |
| Concurrent validity | NR | “The IVIS was significantly correlated with Visual acuity.” We were not able to identify the development study to confirm this, and since no correlation coefficient was reported we graded this NR. |
| Known group validity | NR |  |
| Convergent validity | NR | “The IVIS was significantly correlated with Visual item of the Kurtzke Functional Systems.” We were not able to identify the development study to confirm this, and since no correlation coefficient was reported we graded this NR. |
| Discriminant validity | NR |  |
| Test-retest repeatability | NR |  |
| Responsiveness | NR |  |
| **FAMS, Cella 1996** | | |
| **Property** | **Grade** | **Explanation for grade** |
| Item identification* | NR | “One hundred twenty new items were generated by patients and experts. In addition, 11 questions were drawn from the Fatigue and Spirituality subscales of the FACT Measurement System and the Fatigue Severity Scale developed by the Department of Neurology at the University of Chicago. Four other items were drawn from a review of‘ the MS literature. Thus, 135 new items were generated” Items related to multiple sclerosis without specification of whether sources included or considered optic neuritis. |
| Item selection* | A | Rasch measurement model used “Of the 63 items retained in the principal components analysis, 44 were retained after Rasch measurement criteria were applied to eliminate misfitting items” |
| Acceptability | A | “There is no missing data for the clinical sample” |
| Item targeting | NR |  |
| Internal consistency | A | “Cronbach‘s alpha coefficients are universally high (range 0.82 to 0.961, reflecting homogeneity of the item pool within subscales and for the total score” |
| Response Categories | A | All the categories were ordered, or ordering was obtained after repairing disordered categories, and evenly spaced categories using the “a five point (0 to 4) Likert type response format was used” |
| Dimensionality | A | “The 88-item version of the FAMS, responses of the survey sample (N = 377) were subjected to a principal components analysis with varimax rotation. In this technique, interrelated questions are extracted from the total pool to form “subscales” that depict underlying dimensions or concepts reflected by that collection of questions. Principal components analysis uses “orthogonal” factor rotations, thereby treating every factor as independent of all others. The range of shared variance across all subscale-subscale correlations is 1 to 80% across both samples” |
| Measurement precision | A | Coefficient alpha 0.93 |
| Item fit statistics | NR | Results not reported in paper but “Rasch measurement criteria were applied to eliminate misfitting items. The mean square (MNSQ19) fit statistics, measuring each item’s adherence to the Rasch model restrictions concerning scale uni-dimensionality, was used to determine how well the items fit together to define the underlying variable on a linear scale. The MNSQ has an expected value of 1.0.” |
| Differential item functioning | B | Some items 0.50 to 1.0 logits |
| Targeting | NR |  |
| Concurrent validity | NR | Whilst the authors reported that concurrent validity was assessed, we found no clinical measure against which the instrument was assessed, only many other instruments (convergent validity). |
| Known group validity* | A | “Each group in this breakdown included patients who had been stable for at least 18 months and those who had worsened or had an illness attack within the past 18 months. The second breakdown separated out a subsample of 80 patients (of either relapsing-remitting or progressive type) who had been stable for at least 18 months. In both cases, significant group differentiation was obtained on all the subscales with only one exception.” |
| Convergent validity* | A | “In both samples, the HADS Anxiety scale, HADS Depression scale, MDI Mood scale, and MDI Evaluative scale correlate with the Emotional Well-being and the General Contentment subscales of the FAMS more highly than with any other subscales. In addition, the FAMS Thinking fatigue subscale correlated most strongly with the MDI Vegetative subscale in both samples.” |
| Discriminant validity* | A | “The MCSDS, a measure of social desirability, was given to obtain evidence of divergent validity. No relationship would be expected between quality of life questionnaire and social desirability responses, and none was found.” |
| Test-retest repeatability | A | “Test-retest reliability coefficients are universally high, with coefficients ranging from 0.85 to 0.91” |
| Responsiveness | B | “Instrument tested for responsiveness but statistical significance not reported” |

*note item identification, item selection, known group, convergent and discriminant validity not specific to optic neuritis but multiple sclerosis.
